# Update on Calcium Signaling in Cystic Fibrosis Lung Disease

**DOI:** 10.3389/fphar.2021.581645

**Published:** 2021-03-11

**Authors:** Alessandro Rimessi, Veronica A. M. Vitto, Simone Patergnani, Paolo Pinton

**Affiliations:** ^1^ Department of Medical Sciences and Laboratory for Technologies of Advanced Therapies (LTTA), University of Ferrara, Ferrara, Italy; ^2^ Center of Research for Innovative Therapies in Cystic Fibrosis, University of Ferrara, Ferrara, Italy

**Keywords:** inflammatory disease, calcium signaling, lung disease, inflammation, cystic fibrosis, calcium

## Abstract

Cystic fibrosis (CF) is an autosomal recessive disorder characterized by mutations in the cystic fibrosis transmembrane conductance regulator gene, which causes multifunctional defects that preferentially affect the airways. Abnormal viscosity of mucus secretions, persistent pathogen infections, hyperinflammation, and lung tissue damage compose the classical pathological manifestation referred to as CF lung disease. Among the multifunctional defects associated with defective CFTR, increasing evidence supports the relevant role of perturbed calcium (Ca^2+^) signaling in the pathophysiology of CF lung disease. The Ca^2+^ ion is a critical player in cell functioning and survival. Its intracellular homeostasis is maintained by a fine balance between channels, transporters, and exchangers, mediating the influx and efflux of the ion across the plasma membrane and the intracellular organelles. An abnormal Ca^2+^ profile has been observed in CF cells, including airway epithelial and immune cells, with heavy repercussions on cell function, viability, and susceptibility to pathogens, contributing to proinflammatory overstimulation, organelle dysfunction, oxidative stress, and excessive cytokines release in CF lung. This review discusses the role of Ca^2+^ signaling in CF and how its dysregulation in airway epithelial and immune cells contributes to hyperinflammation in the CF lung. Finally, we provide an outlook on the therapeutic options that target the Ca^2+^ signaling to treat the CF lung disease.

## Introduction

Cystic fibrosis (CF) is a multiorgan genetic disease associated with mutations in the cystic fibrosis transmembrane conductance regulator (CFTR) gene, which preferentially affects the airways causing abnormal infiltration of polymorphonucleated cells, hyperinflammation, and severe lung damage (Riordan, 1993). According to the CF foundation patient registries, more than 70,000 people are living with CF worldwide and about one thousand new cases of CF are diagnosed only in United States each year. The CF is a complex disease, in which the type and severity of symptoms may differ from patient to patient, influencing the individual’s health and the course of disease in a different manner.

The gene’s product is a plasma membrane (PM) ion channel protein located on the apical surface of epithelial cells. Its activation is due by ATP and cAMP-dependent protein kinase A phosphorylations, which extrudes chloride (Cl-) and bicarbonate ions from airway cells (Jacquot et al., 2008). Although CF has long been recognized as an epithelial disease, the channel is expressed also in immune cells (Di et al., 2006; Ng et al., 2016). The most common mutation is the deletion of phenylalanine at position 508 (F508del-CFTR) and the substitution of the amino acid glycine by aspartate at position 551 (G551D-CFTR). The first mutation results in a misfolded protein retained in the endoplasmic reticulum (ER) to be prematurely degraded through the ubiquitin-proteasome pathway. The second is the most prevalent gating mutation, which abolishing the ATP-dependent gating led to a pronounced reduction of channel activity (Pedemonte et al., 2005; Farinha et al., 2013).

Moreover, defective CFTR induces an increased absorption of sodium (Na^+^) coupled with the absence of Cl- secretion. This electrolytic disorder causes the dehydration of periciliary and mucus layers, leading to mucociliary dysfunction and airway mucus plugging (Boucher et al., 1988). An increased susceptibility to pathogen infections, including *Pseudomonas aeruginosa* (*P. aeruginosa*), is associated with defective CFTR, which leads to exaggerated lung inflammatory responses (Bruscia and Bonfield, 2016). In addition, CF patients airways are characterized by abnormal infiltration of neutrophils, which synthesize and release abundant proinflammatory mediators such as interleukin-8 (IL-8) and IL-1β, that contribute to overstimulating the inflammatory responses and worsening the pulmonary injury (Bruscia and Bonfield, 2016).

To improve the lung function, airway clearance techniques and administration of mucus thinner, such as mucolytics, contribute to maintaining the lung clear. Meanwhile, antibiotics and anti-inflammatory drugs fight the infection and consequent hyperinflammation, conditioning the days of CF patients. Recently, a new class of CFTR modulators has been introduced in CF therapy to correct and potentiate the defective CFTR channel. “Correctors” and “potentiators” have garnered much attention in the CF community, although their impact on downstream consequences, such as inflammation, remains debated. The new advances in CF care have drastically ameliorated the quality and duration of life of CF patients.

The calcium ion (Ca^2+^) is a second messenger, which fulfills a plethora of intracellular functions (Giorgi et al., 2018). In addition to its prominent but ambiguous role in energy metabolism and cell death, Ca^2+^ is intimately involved in various cellular processes, such as autophagy and inflammation (Carafoli and Krebs, 2016; Marchi et al., 2018; Patergnani et al., 2020a). Therefore, it is not surprising that altered Ca^2+^ signaling represents a key factor in several inflammatory diseases, including CF. Airway epithelial and immune cells are critically dependent on Ca^2+^ signaling function and integrity. Consequently, perturbations in Ca^2+^ signaling have been observed in CF and are caused by intrinsic defects associated with CFTR deficiency and environmental stress related to recurrent bacterial infections, resulting in an exacerbated inflammatory response that favors lung injury (Ribeiro and Boucher, 2010; Antigny et al., 2011a; Rimessi et al., 2015a).

This review analyzes Ca^2+^ signaling in CF and how its dysregulation contributes to CF lung disease. We summarize the current knowledge and provide an outlook on the therapeutic options to treat the CF lung disease. In particular, we will focus on compounds that target the Ca^2+^ signaling and potentially would lead to adjusting the inflammatory response, thus suggesting new therapeutic strategies for this pulmonary disease.

## Ca^2+^ Signaling in Airway Epithelial and Immune Cells

**FIGURE 1:**
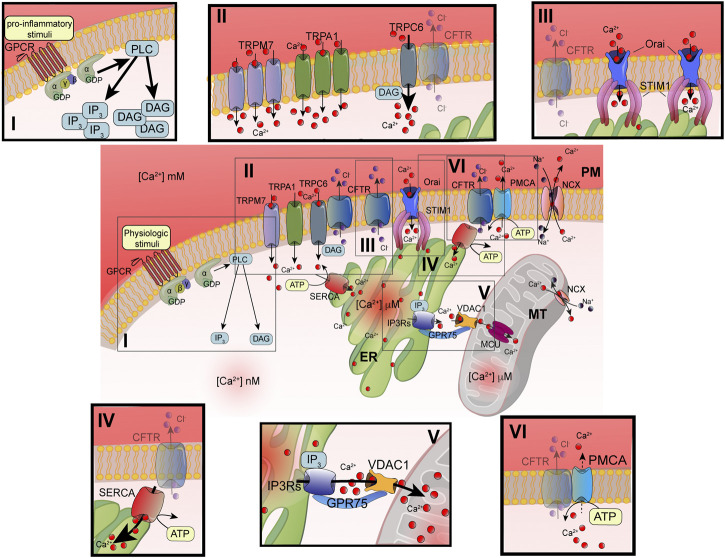
Principal defects in Ca^2+^ signaling associated with defective CFTR channel. Schematic representation of intracellular Ca^2+^ signaling in airway cells. The cellular Ca^2+^ homeostasis is regulated by systems of Ca^2+^-entry and Ca^2+^-efflux located in plasma membrane and organelles. In the inset are reported the principal molecular systems involved in the abnormal intracellular Ca^2+^ signaling associated with defective CFTR channel, where the their dysfunction contributes to physiopathology of CF lung disease: I) increased PLC activity, due to GPCR-dependent overstimulation; II) increased TRP-dependent Ca^2+^-entry, due to enhanced functional activity and/or expression; III) increased Orai insertion with consequent augments in Ca^2+^-influx; IV) increased SERCA activity; V) increased MCU activity; and VI) reduced Ca^2+^-efflux, due to altered PMCA activity. ATP, adenosine triphosphate; Ca^2+^, calcium; CFTR, cystic fibrosis transmembrane conductance regulator; Cl-, chloride; DAG, diacylglycerol; ER, endoplasmic reticulum; GPCR, G protein coupled receptor; GPD, guanosine diphosphate; GRP75, glucose-related protein 75; IP3, inositol 1,4,5-triphosphate; IP3Rs; inositol trisphosphate receptors; MCU, mitochondrial calcium uniporter; NCX, sodium-calcium exchanger; Orai, calcium release activated calcium channel; PLC, phospholipase C; PM, plasma membrane; PMCA, PM-resident Ca^2+^-ATPase; SERCA, ER-resident Ca^2+^-ATPase; STIM1; stromal interaction molecule 1; TRPC, transient receptor potential (TRP) channels; VDAC1, voltage-dependent anion-selective channel 1.

The free cytosolic Ca^2+^ concentration [Ca^2+^]_cyt_ is very low with concentrations in the order of hundreds of nM. This characteristic is guaranteed by a regulated activity of pumps, exchangers, and channels that reside on the PM and by intracellular organelles, such as ER and Golgi, that act as intracellular Ca^2+^ store by accumulating ions in the order of hundreds of μM (Figure 1) (Bootman and Bultynck, 2020).

The advancements in the definition of Ca^2+^ signaling have evidenced the high spatiotemporal complexity and asynchronicity of Ca^2+^ responses. These responses are represented by localized [Ca^2+^]_cyt_ spikes that gradually propagate into the cell as Ca^2+^ waves (Berridge et al., 2003). In nonexcitable cells, such as airway epithelial and immune cells, the [Ca^2+^]_cyt_ spikes are caused by extracellular stimuli, which result in a Ca^2+^-influx from the extracellular space. Agonists, including some proinflammatory and infectious stimuli (e.g., cytokines, bradykinin, prostaglandins, lipopolysaccharides, bacterial flagellin, and pili), are translated in intracellular Ca^2+^ signals through their interaction and subsequent activation of membrane receptors, such as Toll-Like Receptors (TLR) and G protein coupled receptors (GPCRs) linked to phospholipase C (PLC) (Wootten et al., 2018). Activating PLC types, such as PLCβ or γ, catalyzes the hydrolysis of phosphatidylinositol 4,5-bisphosphate (PIP2), giving rise to two second messengers: diacylglycerol (DAG) and 1,4,5-inositol trisphosphate (IP3) (Figure 1i) (Bill and Vines, 2020). DAG may activate transient receptor potential (TRP) canonic (TRPC) channels and classical and novel protein kinase C (PKC) isoforms (Rimessi et al., 2007; Curcic et al., 2019) (Figure 1ii). The DAG-triggering [Ca^2+^]_i_ spikes occur through the direct binding with TRPC channels in PM, in a PKC-independent manner (Curcic et al., 2019). The subfamily of TRPC channels is composed of seven members (TRPC1-7). They are nonselective ion channels permeable to Na^+^ and Ca^2+^. TRPC3, TRPC6, and TRPC7 are the principal contributors of DAG-dependent Ca^2+^-entry in nonexcitable and excitable cells (Figure 1ii).

IP3 induces a transient increase in [Ca^2+^]_cyt_ through the binding with IP3 receptors (IP3Rs), which in turn trigger ER Ca^2+^-release (Foskett et al., 2007) (Figure 1v). The temporal kinetics, the amplitude, and localization of generated Ca^2+^ spikes are strictly dependent on the nature of stimuli. All three IP3Rs induce local Ca^2+^ spikes with similar mean amplitudes, temporal characteristics, and spatial extents (Lock et al., 2018). Normally, IP3Rs are localized in cluster positioned near ER-mitochondria and ER-PM junctions, where the stromal interaction molecule (STIM)/Orai (Ca^2+^-release activated Ca^2+^ modulator 1) complex accumulates Ca^2+^ after the ER store depletion (Thillaiappan et al., 2017; Marchi et al., 2018; Taylor and Machaca, 2019). Intraluminal [Ca^2+^] depletion induces a conformational change in STIM1 and STIM2 isoforms, which translocate in ER-PM interface to bind the PM Orai channel (Figure 1iii). The STIM/Orai complex stimulates the Orai channel opening, giving rise to the store operated Ca^2+^-entry (SOCE) process from the extracellular space. This Ca^2+^-influx mechanism replenishes the ER Ca^2+^ stores and sustains the IP3Rs-dependent phase of increased [Ca^2+^]_cyt_ (Bodnar et al., 2017). A preferential platform for clustering SOCE channels is the caveolae, PM lipid raft microdomains, where Ca^2+^ channels and their regulators are grouped to provide the Ca^2+^-entry also in an IP3R-dependent manner upon the intracellular administration of IP_3_ (Pani et al., 2008; Pulli et al., 2015).

Most of this free intracellular Ca^2+^ is bound by cytosolic proteins or organelles, such as mitochondria and lysosomes, which act as Ca^2+^ buffers (Schwaller, 2020). Another cytosolic Ca^2+^-binding protein involved in the regulation of intracellular Ca^2+^ signaling is Calmodulin. It binds Ca^2+^ through four high affinity binding sites, promoting a direct association and opening of CFTR channel (Bozoky et al., 2017) and regulation of the activity of protein kinases and Ca^2+^-pumps (Villalobo et al., 2018).

**FIGURE 2:**
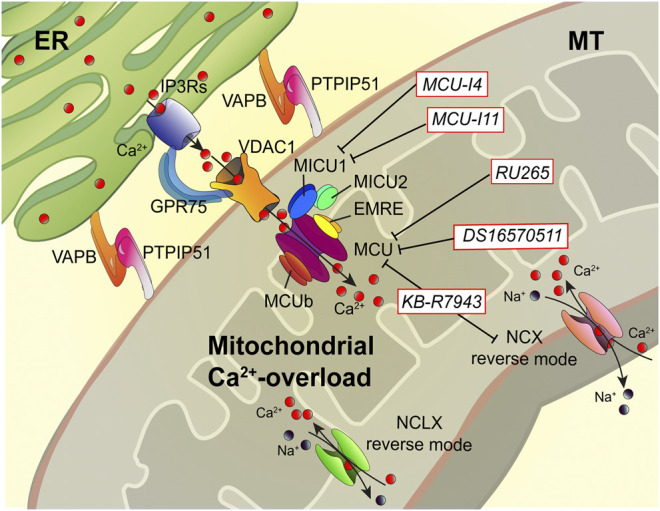
Dampening the mitochondrial Ca^2+^-overload in cystic fibrosis. The dysregulation of Ca^2+^ signaling in CF causes mitochondrial Ca^2+^-overload in airway cells during the recurrent pathogen infections, which leads to organelle dysfunction with repercussion on ROS production and inflammatory responses. The mitochondrial Ca^2+^-overload is mediated by an increased ER-mitochondria Ca^2+^ transfer through the IP3Rs-VDAC-MCU axis due to the stabilization of VAPB-PTPIP51 tethers. Indeed, the increased ENaC-dependent Na^+^ absorption due to defective CFTR in CF could stimulate NCX and NCLX exchangers to work in reverse mode triggering intracellular and mitochondrial Ca^2+^-influx, which may worsen the excessive mitochondrial Ca^2+^-uptake. To dampen the detrimental Ca^2+^ accumulation in matrix, a new class of Ca^2+^ modulator drugs are under investigation; the mitochondrial Ca^2+^-overload inhibitors act on MCU complex and mitochondrial Ca^2+^ exchangers in reverse mode to control the amount of Ca^2+^ imported into the matrix to avoid mitochondrial injury and oxidative stress in CF. Ca^2+^, calcium; EMRE, essential MCU regulator; ER, endoplasmic reticulum; GRP75, glucose-related protein 75; IP3Rs, inositol trisphosphate receptors; MCU, mitochondrial Ca^2+^ uniporter; MICU1, mitochondrial calcium uptake protein 1; MICU2, mitochondrial calcium uptake protein 2; MT, mitochondrion; Na^+^, sodium; NCX, sodium-calcium exchanger; NCLX, mitochondrial Na/Ca exchanger; PTPIP51, protein tyrosine phosphatase interacting protein 51; VAPB, vesicle-associated membrane protein-associated protein B; VDAC1, voltage-dependent anion-selective channel 1.

Mitochondria influence the [Ca^2+^]_cyt_ by inducing a transient sequestering of the Ca^2+^ released at the ER-mitochondria interfaces (Marchi et al., 2018). In these intimate and dynamic regions between ER and mitochondrial outer membranes (OMMs), called also mitochondria-associated ER membranes (MAMs), a series of specialized molecular bridges control the frequency of interactions, the size and the spacing between the organelles, and changing at front of cellular and functional requests (Simmen and Herrera-Cruz, 2018; Bootman and Bultynck, 2020). Thus, the mitochondrial Ca^2+^-transfer is firstly favored by the distance from ER and number of mitochondria involved in these interorganelle couplings and secondly by the negative membrane potential in mitochondrial matrix generated by the respiratory chain (Csordas et al., 2010; Rimessi et al., 2015b). The Ca^2+^ is then transmitted into the matrix by the mitochondrial Ca^2+^ uniporter (MCU) (Figure 1v) (Baughman et al., 2011; De Stefani et al., 2011). MCU is a transmembrane protein of inner mitochondrial membrane (IMM), assembled as tetramer, which forms a high selective Ca^2+^-channel with low affinity for the ion. Its activity is regulated by the EF-hand-containing Ca^2+^-binding proteins mitochondrial calcium uptake 1 (MICU1) and MICU2, which together with other forming-channel elements, such as MCUb and essential MCU regulator (EMRE), constitute the MCU complex (Figure 2) (Marchi and Pinton, 2014). However, before reaching the IMM, Ca^2+^ must cross the OMM mediating the voltage-dependent anion channels (VDACs), involved also in the transport of adenosines (ATP, ADP) and metabolites, including pyruvate and malate (Shoshan-Barmatz et al., 2010). Three different isoforms of VDAC have been identified: VDAC1-3. Among them, VDAC1 has Ca^2+^ binding sites and is highly Ca^2+^ permeable and modulates the accessibility of ion to the mitochondrial intermembrane space (IMS) (Gincel et al., 2001). The mitochondrial Ca^2+^ is released more slowly back into the cytosol by Na^+^-dependent exchange mechanisms in excitable and nonexcitable cells via Na^+^/Ca^2+^ exchanger (NCX) and Na^+^/Ca^2+^/Li^+^ exchanger (NCLX) (Khananshvili, 2014; Kostic and Sekler, 2019). NCX, located on the OMM, may operate either in forward mode, extruding one Ca^2+^ ion from mitochondrial intermembrane space *vs* three Na^+^ ions in influx from cytosol, or in reverse mode, exchanging Ca^2+^-influx/Na^+^-efflux. NCLX, located on the IMM, transports Ca^2+^ outside the matrix in exchange of either Na^+^ or Li^+^ at similar rates (Figures 1, 2). In nonexcitable cells, the mitochondrial Ca^2+^ is also extruded by H^+^/Ca^2+^ exchanger (Nishizawa et al., 2013).

However, after removing the stimulus, the [Ca^2+^]_cyt_ is rapidly lowered through the activation of Ca^2+^-ATPase pumps located on the PM and ER, respectively (Figure 1iv–vi). PM Ca^2+^-ATPase (PMCA) push out Ca^2+^ from cell while sarco/endoplasmic reticulum Ca^2+^-ATPase (SERCA) pumps Ca^2+^ back into the ER (Domi et al., 2007). These pumps are P-type ATPase, which exchange one (PMCA) or two (SERCA) Ca^2+^ ions for hydrolyzed ATP (Strehler and Treiman, 2004; Chen et al., 2020a). PMCA presents a high Ca^2+^-affinity but low Ca^2+^-transporting rate. In support of the PM Na^+^/Ca^2+^ exchangers, a second Ca^2+^-efflux system with low Ca^2+^-affinity but high Ca^2+^-transporting rate contributes to clamping the [Ca^2+^]_cyt_ at homeostatic levels.

## Abnormal Ca^2+^ Signaling in Cystic Fibrosis and Physiopathological Consequences

To date, increasing evidence highlights the importance of perturbed Ca^2+^ signaling in CF lung disease’s physiopathology. The abnormal Ca^2+^ profile observed in CF airway epithelial and immune cells is initially due to intrinsic defects associated with mutated CFTR. It is sustained successively by recurrent pathogen infections and by overstimulation of released proinflammatory mediators, resulting in detrimental lung inflammation (Ribeiro, 2006; Antigny et al., 2011a).

### Defective CFTR and Ca^2+^ Signaling

Ca^2+^ signals have key roles in the CFTR channel function and in airway immune responses, which are perturbed in CF. Ca^2+^ signaling controls the CFTR protein expression levels and internalization (Bargon et al., 1992; Patel et al., 2019), while at level of airways, it regulates ciliary beating and secretion of fluid and antimicrobial agents (Salathe, 2007; Waterer, 2012; Lee and Foskett, 2014).

In CF, Ca^2+^ exacerbates the airway inflammatory responses (Figure 1i). Its dysregulation has been observed in several human CF patient-derived primary cells: airway epithelial cells (Rimessi et al., 2015a), bronchial goblet cells (Roomans, 1986), skin fibroblasts (Shapiro et al., 1978), kidney cells (Katz et al., 1988), and immune cells such as leukocytes, neutrophils, and lymphocytes (Banschbach et al., 1978; Waller et al., 1984; Robledo-Avila et al., 2018). In all of them, the [Ca^2+^]_cyt_ was increased compared to non-CF cells, demonstrating that functional CFTR regulates the Ca^2+^ homeostasis conditioning and in turn the interorganelle Ca^2+^-transfer evoked by stimuli (Rimessi et al., 2015a).

The increased [Ca^2+^]_cyt_ in CF airways results from an enhanced Ca^2+^-entry mediated by PM Ca^2+^-channels and reduced Ca^2+^-efflux operated by PMCA, influencing the Ca^2+^ accumulation into the stores (Figures 1i,ii,vi) (Philippe et al., 2015). In particular, the TRP channel family is involved in abnormal Ca^2+^-entry in CF airway cells (reviewed in (Grebert et al., 2019)) (Figure 1ii). TRPC6-mediated Ca^2+^-influx was increased in F508del-CFTR and G551D-CFTR airway cells with respect to non-CF cells when exposed to 1-oleoyl-sn-glycerol, a synthetic, cell-permeable compound analogous to DAG, used as an activator of PKC (Antigny et al., 2011b). No difference in TRPC6 expression justified the discrepancy, but it has been observed that the physical interaction between WT-CFTR and TRPC6 channel downregulated the Ca^2+^-influx in airway epithelial cells, suggesting that the lacked or reduced CFTR expression in PM in CF cells perturbs the functional coupling between the two channels favoring the abnormal Ca^2+^-entry (Antigny et al., 2011b). However, specific TRPC6-silencing or the CFTR-corrector agent’s administration, VX-770, reduced the abnormal [Ca^2+^]_cyt_ increment and IL-8 release, indicating that defective CFTR worsens the inflammatory response affecting the TRPC6 activity (Vachel et al., 2013). Similar effects were also observed for TRP vanilloid 4 and 6 channels (TRPV4 and TRPV6), respectively. The intracellular Ca^2+^ elevation by TRPV4 stimulation led to CFTR channel activation in bronchial epithelial cells in physiological condition (Genovese et al., 2019). The increased Ca^2+^-influx and the inflammatory response in CF were attenuated by genetic manipulation of TRPV channels and by low temperature-mediated F508del-CFTR rescue in CFTR-deficient cells, mediating reduction of [Ca^2+^]_cyt_ mitigated release of IL-8, prostaglandin E2 and keratinocyte chemo-attractant (Vachel et al., 2015; Henry et al., 2016).

In addition, an increased expression in PM of nonselective Ca^2+^ channel TRP subfamily M member 7 (TRPM7) has been observed in both F508del-CFTR- and G551D-CFTR-expressing cells, resulting in higher Ca^2+^-influx than WT-CFTR-expressing cells (Huguet et al., 2016). A similar increase in expression of TRP ankyrin subtype 1 channel (TRPA1) was observed in human CF respiratory epithelium and in CF airway cells. Here, TRPA1 pharmacological modulation controls the transcription and release of several proinflammatory mediators, including IL-8 and IL-1β, in a Ca^2+^-dependent manner (Prandini et al., 2016). Orai expression resulted in enhanced CF airway cells with a consequent increase in Ca^2+^-entry and release of IL-8 (Figure 1iii) (Balghi et al., 2011). The STIM1 migration and Orai activity do not occur only during ER depletion but may also occur upon mitochondrial Ca^2+^-efflux, as demonstrated in basophilic leukemia, embryonic fibroblast, and kidney cells, with dangerous repercussions on inflammation (Singaravelu et al., 2011; Delmotte et al., 2012).

Abnormal Ca^2+^-entry is a primary signal associated with defective CFTR that conditions the ER Ca^2+^-accumulation and mitochondrial Ca^2+^-overload elicited in CF cells, already due also to increased SERCA activity (Figure 1vi) (Philippe et al., 2015). The functional interaction between WT-CFTR with SERCA2b and PMCA observed in non-CF cells is partially lost in CF cells, due to ER retention of F508del-CFTR mutant, with several implications on the ER and PM Ca^2+^ channel activities, which potentiate the intraluminal Ca^2+^ accumulation (Norez et al., 2006). A study identified Calumenin as interactor of CFTR channel, a Ca^2+^-binding protein located primarily in the ER able to regulate ER Ca^2+^ homeostasis, interacting with SERCA and the ER Ca^2+^ channel ryanodine receptors (RyRs) (Vorum et al., 1999; Jung et al., 2006; Sahoo et al., 2009; Teng et al., 2012). The binding between Calumenin and CFTR increased when the channel presented the prevalent gating mutation G551D-CFTR (Teng et al., 2012). Indeed, Calumenin contributed to the ER retention of mutated F508del-CFTR channel, if silenced PM expression and activity of mutated CFTR channel were restored in bronchial epithelial cells (Philippe et al., 2017).

Mitochondrial Ca^2+^-uptake was increased in primary CF airway epithelial cells, skin fibroblasts, and lymphocytes (Feigal et al., 1982; Waller et al., 1984; Rimessi et al., 2015a), mediated by a greater ER Ca^2+^-transfer and by perturbed respiratory activity, which stimulated mitochondrial ROS production, mitochondrial injury, and release of mitochondrial damage-associated molecular patterns in CF lung (Figure 1v) (Feigal et al., 1982; Antigny et al., 2011a). Severe mitochondrial dysfunctions in basal condition were restricted to human F508del-CFTR tracheal gland CF-KM4 cell clone. In this cell clone, authors found a reduced mitochondrial Ca^2+^ uptake consequence of mitochondrial membrane depolarization and perturbed network, resulting from an altered mitochondrial physiology (Antigny et al., 2009).

At demonstration of the presence of an abnormal mitochondrial Ca^2+^ accumulation, different studies unveiled that, by using corrector agents, such as VX-770 and VX-809, or by rescuing functional F508del-CFTR, it is possible to normalize the mitochondrial [Ca^2+^] levels with beneficial repercussions on oxidative stress and the levels of proinflammatory mediators released, such as IL-8 and the inflammasome-dependent cytokine IL-1β (Vachel et al., 2013; Rimessi et al., 2015a; Philippe et al., 2015).

### Ca^2+^ Signaling in CF Infection and Inflammation

Airway epithelial cells respond to pathogens, such as *P. aeruginosa*, through Ca^2+^-dependent mechanisms to produce proinflammatory mediators to initiate the inflammatory response (Ratner et al., 2001; Fu et al., 2007). Different bacterial constituents, including LPS, promote TRP-dependent Ca^2+^-entry and ER Ca^2+^-release via IP3Rs (Buyck et al., 2013). Pili and flagellin interact with TLR2, TLR4, TLR5, and Asialo ganglio-N-tetraosylceramide (Asialo GM1) receptor and induce the expression of IL-8 mediating the activation of NF-kB, which is also phosphorylated by Ca^2+^-dependent PKC isoforms α and β that respond to intracellular Ca^2+^ flux following IP3R-dependent ER Ca^2+^-release (Pinton et al., 2004; Asehnoune et al., 2005; Chun and Prince, 2006). This Ca^2+^-dependent activation of NF-kB is also sustained by Asialo GM1-dependent nucleotides released from airway epithelial cells that interact with flagellin, which binds the purinergic P2Y receptors active intracellular Ca^2+^-signaling (Mcnamara et al., 2006; Billet and Hanrahan, 2013).

In CF, the airway epithelial cells respond to the recurrent infection generating abnormal Ca^2+^ mobilization to produce many cytokines and chemokines, useful to recruit leukocytes to contrast the accumulated bacteria in the airways. This overstimulation determines a Ca^2+^-dependent hyperinflammation phenotype. CF airway epithelial cells result in hyperresponsiveness to pathogens due to the increased [Ca^2+^]_cyt_, which contributes to 1) ER expansion and increased intraluminal [Ca^2+^]; 2) mitochondrial Ca^2+^-overload and consequent organelle dysfunction; and 3) an exuberant and more prolonged NF-kB activation, priming the cells to excessive expression and release of proinflammatory mediators (Ribeiro et al., 2005; Tabary et al., 2006; Rimessi et al., 2015a; Rimessi et al., 2020).

The ER Ca^2+^ store expansion in CF airway cells is due to activation of the inositol-requiring enzyme 1 (IRE1)/X-box binding 1 (XBP-1) pathway, which is not the consequence of misfolded CFTR. Reductions in ER Ca^2+^-release in CF samples were also obtained by correcting F508del-CFTR trafficking by miglustat (N-butyldeoxynojirimycin) or low temperature (27°C) (Antigny et al., 2008a; Antigny et al., 2008b). Antigny et al. demonstrated that the abnormal ER Ca^2+^-release in CF gland CF-KM4 clone was due to a dysfunctional IP3Rs, consequence of ER retention of mutant CFTR channel (Antigny et al., 2008b). The importance of IP3Rs in CF was then confirmed by Martins et al. in nasal epithelial cells, where it was demonstrated that the ER retention of F508del-CFTR determined a functional interference with IP3-receptor binding protein IRBIT, which suppresses the activation of IP3Rs by competing with IP3 for binding to the ligand-binding domain (Ando et al., 2006; Martins et al., 2011).

However, the increased flux of newly synthesized proinflammatory mediators into the ER in response to recurrent infections contributes to ER expansion (Ribeiro and Lubamba, 2017). Besides, changes in the intracellular redistribution of ER have been observed in response to pathogens. In this case, the ER moved to the apical level of polarized CF airway epithelial cells to facilitate the GPCR-induced Ca^2+^ responses (Ribeiro et al., 2005).

Recently, Rimessi et al. demonstrated that *P. aeruginosa* infection increases ER-mitochondria juxtapositions in CF airway epithelial cells by stabilizing the ER protein vesicle-associated membrane protein-associated protein B (VAPB) and the outer mitochondrial membrane protein tyrosine phosphatase interacting protein 51 (PTPIP51) tethers, favoring the mitochondrial Ca^2+^ transfer via MCU (Figure 2) (Rimessi et al., 2020). This led to mitochondrial membrane potential loss, ROS production, and organelle dysfunction, inducing persistent mitochondrial Unfolding Protein Response (UPR^mt^) and NLRP3 inflammasome activation. In turn, these processes downregulated the selective autophagic responses, mitophagy, and xenophagy, resulting in augmented pathogen survival and worsening of inflammatory response (Rimessi et al., 2015a; Rimessi et al., 2020). Thus, the mitochondrial Ca^2+^-overload in CF airway cells plays a crucial role in the evolution of CF pulmonary inflammation. Preventing the mitochondrial Ca^2+^-overload, via MCU inhibition, the *P. aeruginosa*-dependent mitochondrial dysfunction was abrogated in CF airway cells, while the selective autophagic responses were rectified (Rimessi et al., 2020).

A higher predisposition of CF airway cells to NLRP3 inflammasome activation is also due to the dysregulation of ENaC-dependent Na^+^-influx associated with defective CFTR, which predisposes the cells to K^+^-efflux, a further activating signal to NLRP3 inflammasome (Scambler et al., 2019).

Excessive Ca^2+^-dependent IL-8 secretion is critical for CF lung disease development and is responsible for abundant neutrophil recruitment into the lung. IL-8 production is 13-fold higher in CF bronchial cells than non-CF cells and occurs through persistent and prolonged NF-kB activation (Tabary et al., 2000). A Single Nucleotide Polymorphisms (SNP) genetic study from a panel of 135 genes implicated in the signal transduction for neutrophil recruitment, identifying PLC beta-3 (PLCB3) gene on top of the rank, involved in the excessive expression and release of IL-8 during *P. aeruginosa* infection in F508del-CFTR patients (Bezzerri et al., 2011). The c.2534C > T (p.S845L) PLCB3 is a loss-of-function variant associated with a mild progression of CF lung disease, where its inability to trigger intracellular Ca^2+^ transient limited the activation of Ca^2+^-dependent PKCs and NF-kB, reducing the *P. aeruginosa*-dependent induction of IL-8 transcription and protein release in primary CF patient-derived airway epithelial cells (Rimessi et al., 2018). Similar effects have been observed by inhibiting the Ca^2+^-dependent PKCα isoform with β-sitosterol, which blocked the *P. aeruginosa*-triggering IL-8 induction and release in CF airway cells (Lampronti et al., 2017).

The abnormal intracellular Ca^2+^ signaling associated with defective CFTR also implies the abundant neutrophils recruited into CF lung during the recurrent bacterial infections. An increased [Ca^2+^]_cyt_ has been measured in human CF neutrophils compared to non-CF, which correlates with a reduced antimicrobial killing capacity due to diminished NADPH oxidase response and impaired secretion of neutrophil extracellular traps (Robledo-Avila et al., 2018). The higher [Ca^2+^]_cyt_ in CF neutrophils is sustained by increased Ca^2+^-entry via TRP channels, especially TRPM2 and TRPM7 channels, which when inhibited with 2-aminoethoxydiphenylborane restored the antimicrobial response of CF neutrophils during infection, preventing the intracellular Ca^2+^-overloading (Heiner et al., 2005; Park et al., 2014; Robledo-Avila et al., 2018).

## Targeting Ca^2+^ Signaling as Alternative Anti-Inflammatory Approach

Understanding the molecular mechanisms that induce hyperinflammation in CF lung through the Ca^2+^ signaling impairment helps to identify new and alternative therapeutic targets to treat the CF lung disease. Thus, pharmacological Ca^2+^ signaling-targeting agents aim to control the increased [Ca^2+^]_cyt_, ER, and mitochondrial Ca^2+^-overload in CF airway epithelial and immune cells, which may be considered a new class of anti-inflammatory drugs to prevent the hyperinflammatory response in CF.

### TRP Channel Inhibitors

TRPA1 results in a druggable target to control the excessive inflammation in CF. The selective inhibition of TRPA1, by HC03 or A96 antagonist, reduced the induction and release of IL-8, IL-1β, and TNFα in CF patient-derived airway cells during *P. aeruginosa* infection (Prandini et al., 2016). Recently, the new antagonists of TRPA1 ODM-108, CB-625, CB-189625, and HX-100, which were under investigation in different phases of clinical trials to treat asthma and chronic obstructive pulmonary disease, have been discontinued for pharmacokinetics reasons (Chen and Terrett, 2020). In addition, GRC-17536, which had obtained promising results in preclinical studies on inflammatory animal models, has been suspended from clinical trials (Preti et al., 2012; Mukhopadhyay et al., 2014; Chen and Terrett, 2020). Also, TRPV4 plays a role in the excessive Ca^2+^-entry in CF. A new inhibitor derived from the TRPV4-inhibitor GSK205, called compound 16-8, has been developed to target simultaneously TRPV4 and TRPA1 channel to block the Ca^2+^-influx, showing the potentially advantageous property to apply to CF hyperinflammation (Kanju et al., 2016).

TRPC6 is another druggable target to counteract the increased [Ca^2+^]_cyt_ in CF. BI-749327 is an orally selective TRPC6-inhibitor used to suppress renal inflammatory cell infiltration and fibrosis, ameliorating renal stress-induced disease (Lin et al., 2019). SAR7334 was initially identified as a potent TRPC6-inhibitor, but this agent may inhibit the Ca^2+^-influx mediated by TRPC3 and TRPC7. SAR7334 attenuated the IL-6 and IL-8 release in human bronchial epithelial cells exposed to ozone (O_3_), protecting from the O_3_-induced airway inflammatory response *in vivo* (Chen et al., 2020b). The nonselectivity of TRPC antagonist SKF-96365 limits the clinical usefulness of this drug although it reduced the LPS-dependent secretion of TNFα and IL-6 in microglia. In contrast, carvacrol, a phenolic monoterpene, through the selective inhibition of TRPM7, reduced the oversecretion of proinflammatory cytokines such as IL-1β, TNFα, and IL-6 in endotoxemic rats (Heo et al., 2015; Gatica et al., 2019).

### PLC Inhibitors

PLC activity concurs to abnormal Ca^2+^ signaling in CF inducing the TRP channels activation and ER Ca^2+^-release through the recurrent generation of DAG and IP3. Thus, the inhibition of PLC may contribute to attenuating the hyperinflammatory response in CF, reducing the Ca^2+^-entry and the mitochondrial Ca^2+^-overload limiting TRP channels activation and ER-mitochondria Ca^2+^-transfer, respectively. Edelfosine was the first PLC inhibitor identified, decreasing the ER Ca^2+^-release in tumor cells, but its cytotoxicity limits the clinical usefulness (Berkovic, 1998). U73122 is another PLC inhibitor and is used as an anti-inflammatory agent in different pathological contexts. U73122 may inhibit the LPS- or influenza A virus-induced expression of cytokines IL-1β and TNFα in human promonocyte U937 cells and in mouse primary peritoneal macrophages (Zhu et al., 2015; Zhu et al., 2016). Unfortunately, accumulating reports highlight off-target effects of U73122, including SERCA, Kir3, and Ca^2+^-activated K^+^ channels, calling into question its selectivity (Klose et al., 2008; Hollywood et al., 2010). A high-throughput analysis has been performed on 6,280 compounds, identifying three putative PLC activity inhibitors. Unfortunately, these compounds are not optimal to inhibit cellular PLC activity because they present a reduced cell permeability and a limited potency. Nevertheless, these compounds may be useful for development of new drugs to interrupt the abnormal signaling cascades controlled by PLCs, for the treatment of human diseases, including cancer (Huang et al., 2013).

### Mitochondrial Ca^2+^-Overload Inhibitors

The mitochondrial Ca^2+^-overload inhibitors are pharmacological agents that by preventing the detrimental Ca^2+^ accumulation in the matrix reduce mitochondrial injury and oxidative stress, which are necessary to amplify the proinflammatory signals and activate NLRP3 inflammasome in CF lung disease (Figure 2) (Rimessi et al., 2015a; Rimessi et al., 2020). Limiting the excessive Ca^2+^ transport into mitochondria, via MCU, represents the first therapeutic approach with promising results both *in vitro* and *in vivo* in CF (Rimessi et al., 2015a; Rimessi et al., 2020). The MCU inhibitor, KB-R7943, reduced lung inflammation in *P. aeruginosa*-inoculated CF mice. The treated mice showed a reduction of interstitial inflammatory infiltrate with a general reduction in the congestion of lung parenchyma and restoration at the level of interalveolar septa of inflammatory infiltrate clearing conditions (Rimessi et al., 2020). Indeed, KB-R7943 rectified the unbalanced selective autophagic activities, thus restoring mitochondrial quality control and bacterial clearance capacity in CF airway cells. KB-R7943, designed to inhibit NCX in reverse mode, is the first cell-permeable MCU inhibitor available (Iwamoto et al., 1996; Santo-Domingo et al., 2007). Its nonspecificity and toxicity at high concentrations have limited its clinical usefulness, but its multitasking activity in CF on inflammation, mitochondrial stress response, and autophagy could represent an important starting point to develop new drugs to treat CF (Figure 2). A new class of selective and cell-permeable MCU inhibitors is now commercially available, namely, Ru265 and DS16570511, until now used only *in vitro*, but could have therapeutic implications in CF in the future (Kon et al., 2017; Woods et al., 2019).

The abnormal mitochondrial Ca^2+^-uptake in CF may be controlled through MCU and mitochondrial NCX reverse targeting. The increased ENaC-dependent Na^+^ absorption in CF could stimulate NCX and NCLX exchanger to work in reverse mode, triggering intracellular and mitochondrial Ca^2+^-influx (Berdiev et al., 2009; Verkhratsky et al., 2018).

As an alternative, mitochondrial Ca^2+^-overload may be prevented by inhibiting MICU1 activity (Figure 2). MCU complex comprises the pore-forming MCU protein, EMRE, and the gatekeepers MICU1 and MICU2, which regulate the MCU activity sensing the changes in [Ca^2+^]_cyt_. Recently, two new pharmacological MICU1 inhibitors have been developed, MCU-i4 and MCU-i11, both blocking the IP3-dependent mitochondrial Ca^2+^-uptake, maintaining the gatekeeping role of their target (Di Marco et al., 2020). Hence, the MICU1 inhibitors should allow a greater fine-tuning modulation of mitochondrial Ca^2+^-uptake than the known MCU inhibitors. This aspect could be relevant to treat the hyperinflammation in CF lung disease. The last strategy feasible should be to activate the mitochondrial Ca^2+^-efflux mechanisms, but selective activators or inducer have not been identified.

Some potential pitfalls could emerge about mitochondrial Ca^2+^-overload inhibitors, concerning the safety and biochemical stability of new molecules *in vivo*, the limitations in current knowledge (being recently discovered), and the long-term efficacy of mitochondrial Ca^2+^ signaling modulation that would be expected to alter the cell metabolism. However, no differences in basal oxygen consumption have been observed between WT and MCU-KO mice, suggesting that basal metabolism was not markedly altered in absence of mitochondrial Ca^2+^ signal (Pan et al., 2013; Murphy et al., 2014).

The enhancement of mitochondrial quality control through the pharmacological modulation of mitochondrial Ca^2+^ signaling is emerging as alternative anti-inflammatory strategy for the prevention or treatment of mitochondrial-associated disorders, such as CF (Patergnani et al., 2020b). Drugs that directly affect mitochondria, and thus mitochondrial Ca^2+^ signaling, have been recently used as a main mode of action to treat diseases, such as type 2 diabetes and cancer, as well as upregulating the immune system to clear infection with promising success (Stoker et al., 2019).

## Conclusion

Substantial evidence supports the theory that the dysregulation in Ca^2+^ signaling associated with defective CFTR is essential for the development of the hyperinflammatory phenotype observed in CF lungs. This dysregulation involves different cells leading to multifunctional defects in CF patients. Both airway epithelial and immune cells are affected, with heavy repercussions on cell function, viability, and susceptibility to pathogens, which contribute significantly to the degeneration of pathological conditions of CF lung disease. Targeting the abnormal Ca^2+^ signaling in CF represents a new and attractive therapeutic strategy useful for reducing the proinflammatory overstimulation, organelle dysfunction, oxidative stress, and cytokines release in the CF lung.

“Correctors” and “potentiators” that are the new frontier in CF therapy, despite their positive impact in the CF community, are debated about their downstream consequences, in particular on inflammation. Evidence shows that *P. aeruginosa* burden decreased in the first six months of modulator therapy but rebounded thereafter, increasing the inflammatory response (Hisert et al., 2017). In addition, *P. aeruginosa* has been shown to directly reduce the apical membrane expression of rescued ΔF508CFTR and the following chloride secretion (Rubino et al., 2014). These facts are relevant since the presence of *P. aeruginosa* infection could *per se* render the recent CF therapies less effective. Therefore, alternative approaches aimed at activating early anti-inflammatory pathways to prevent organ damage before patients become symptomatic are needed. To date, new class of alternative anti-inflammatory drugs is emerging to prevent the inflammatory signal amplification and tissue degeneration related to chronic inflammation in CF. The cure of the lung pathology of CF patients will rely on the association of drugs acting as “correctors” and “potentiators” on the mutated CFTR protein together with novel anti-inflammatory drugs, such as the Ca^2+^-modulators, and more active antibacterial drugs against *P. aeruginosa*.
